# Supplementary material for: Are carbon emissions trading and green financial instruments synergistic? -Comprehensive quantitative research based on content analysis
Source: PLoS One. 2024 Mar 7;19(3):e0298601. doi: 10.1371/journal.pone.0298601 (PMC10919601; doi:10.1371/journal.pone.0298601)
Supplement: S1 Data — (ZIP) [file pone.0298601.s001.zip › Data/地级市Z_F工作报告.zip!/┴╔─■/╙¬┐┌╩╨/└┤╫╘╣1⁄2╓┌║┼í╛┬φ┐╦╩2╛▌═°í┐_2017.docx]

各位代表：

现在，我代表市政府向大会报告工作，请予审议，并请市政协各位委员提出意见。

一、2016年工作回顾

过去的一年，是营口经济社会发展稳中求进的一年，是为顺利实施“十三五”规划、全面建成小康社会夯实基础的一年。一年来，在省委、省政府和市委的正确领导下，在市人大、市政协的监督支持下，市政府认真学习贯彻习近平总书记系列重要讲话精神，以“五大发展理念”为统领，以“四个着力”为主线，积极应对严峻复杂的国内外经济形势，始终坚持以经济建设为中心，适应把握引领新常态，求真务实，真抓实干，全市经济社会发展取得了新进展新进步。

预计全年地区生产总值完成1300亿元，固定资产投资410亿元，一般公共预算收入105.2亿元，社会消费品零售总额506.3亿元，城镇常住居民人均可支配收入32285元，农村常住居民人均可支配收入14517元，城镇登记失业率2.98%。

过去的一年，面对巨大的困难和挑战，全市上下迎难而上，奋力前行，主要做了以下六方面工作：

（一）全力以赴稳增长，经济发展基础不断夯实

政策效应逐步显现。坚持把抢抓政策机遇作为振兴发展的重要抓手，认真贯彻落实党中央、国务院和省委、省政府各项决策部署，积极对接国家“一带一路”、新一轮东北振兴等重大战略，逐项分解落实中央7号文件、国务院62号文件等政策举措，确保各项政策在营口落地生根。全年争取国家、省各类资金90亿元。我市被国家列入东北地区民营经济发展改革示范市。

发展动力加速转换。牢固树立抓创新就是抓发展、谋创新就是谋未来的理念，深入实施创新驱动发展战略。全年新建省级产业专业技术创新平台3个、工程技术研究中心和重点实验室各1家，新认定国家级高新技术企业6户。中小企业创业园区晋升国家级小微型企业创业创新示范基地，营口众创空间、九州创客工坊及华讯众创空间被认定为国家级众创空间，创新创业活力有效释放。

发展潜力日益提升。全面落实省项目年部署，狠抓招商引资上项目，着力解决项目建设“最后一公里”问题，积极扩大有效投资，全年开工亿元以上项目270个。投资437亿元的忠旺集团特种车辆等8个产业链延伸项目有序推进，投资100亿元的环渤海大数据产业园等一批重点项目开工。积极盘活存量，清理整顿环保违规项目1440个，整合利用闲置行政办公用房15万平方米、闲置土地516公顷，盘活停产半停产企业60户。不断扩大消费需求和供给，“互联网+消费”新模式不断壮大，鲅鱼圈万达广场、北海亚特兰蒂斯海乐园等消费载体投入运营。加大出口企业服务扶持力度，全年外贸出口完成37亿美元，新增外贸出口企业192户。

（二）多措并举调结构，转型升级取得新成效

现代农业稳步发展。围绕推动农业增效、农民增收，加快转变农业发展方式。农业综合生产能力不断增强，粮食产量达到73.5万吨，连续11年喜获丰收。农业产业化迈出新步伐，全年新建千万元以上产业化项目10个，新增规范农民专业合作社108家、省级示范家庭农场14个。农村特色产业初具规模，盖州市被确定为国家农村一二三产业融合发展试点县。农村环境集中综合整治取得阶段性成果，省批准的66个美丽乡村建设初见成效。农村综合改革稳步推进，完成耕地确权登记颁证100万亩，新增土地流转3万亩。

工业经济持续优化升级。加快调整产业结构、产品结构，全面落实《中国制造2025营口行动纲要》，华润金属公司智能车间获评国家智能制造试点示范项目。深入推进“千企转型升级行动”，300户企业实现转型发展。开展专项帮扶行动，精准施策，解决企业问题300余个。大力支持民营经济发展，民营经济增加值达到1000亿元。推动工业产业集群做大做强，七大主导产业主营业务收入实现1750亿元，产业集聚度和丰厚度进一步提高。

服务业支撑作用明显增强。坚持围绕优势抓三产，现代服务业发展水平不断提升。全市9个省级现代服务业集聚区主营业务收入增长14.3%。商贸流通业转型升级步伐加快，辽宁北方商品交易中心等4家交易平台累计实现交易额1879亿元，阿里巴巴（营口）产业带入驻企业突破1000家。现代物流业快速发展，全市物流相关企业超过5000家。旅游业发展实现新突破，21个亿元以上重点旅游项目完成投资50.8亿元。全年旅游总收入实现210.6亿元，增长13.8%。营口荣获“2016中国最具魅力宜居宜业宜游旅游城市”奖。

（三）锐意进取抓改革，市场发展活力有效释放

供给侧结构性改革取得初步成效。全面落实“三去一降一补”重点任务，大力化解过剩产能，淘汰镁质材料落后产能90万吨。有序消化房地产库存，省下达的178万平方米去库存任务全部完成。积极降低企业杠杆率，帮助企业拓宽融资渠道，新引进金融机构5家，新增融资总量572亿元。降低实体经济企业成本，全年为企业减负16亿元；争取直购电68亿千瓦时，节约企业成本9.5亿元。突出重点补短板，改造棚户区住房4897套，安置回迁户13510户，改造农村危房975户；全年完成移民350户，稳定脱贫12852人，贫困村摘帽10个。

“放、管、服”改革扎实推进。围绕破除影响发展的体制机制障碍，更加突出简政放权，清理市本级行政审批等事项112项，在全国率先出台了取消行政审批等事项的后续监管措施清单。组建市行政审批局，对市本级29个行政部门、312项行政许可权集中管理。推进商事制度改革，新增市场主体2.8万户。营商环境持续改善，营口综合信用指数居全省前列，诚信品牌正在形成。

国资国企改革迈出新步伐。进一步完善11家企业集团监管体系。营口港务集团、营口交通物流集团等企业通过股权多元化改革引进各类投资16.5亿元，实现了零的突破。沿海公交公司、海为盐业公司整合划转和供电、通信“三供一业”分离移交工作全面完成，国企发展活力进一步增强。

事业单位改革稳妥推进。全面启动事业单位分类改革工作，90余家市属事业单位列入改革范围，制定出台了事业单位转企改制实施意见。我市被中央编办确定为全省唯一行政类事业单位改革试点市。

财政和投融资体制改革取得新进展。规范政府债务管理，全年置换政府债务286.2亿元，减少利息支出6.4亿元，政府债务风险总体可控。积极推广政府与社会资本合作模式，投资28.5亿元的沿海产业基地基础设施整体运营项目顺利实施。设立中小企业可持续发展基金、股权（创业）投资引导基金。港口金融创新试验区建设稳步推进，辽宁北方金融资产交易中心实现交易额1800亿元。新增上市和资本市场挂牌企业8家。

公车、价格改革顺利实施。全面完成公务用车制度改革，全市公务用车减少2146辆，节支率达到12.1%。完善定价清单制度，实现了清单之外无定价。

（四）精准发力促开放，外向型经济发展水平稳步提升

大通道建设成效显著。依托区位、港口优势，深度融入“一带一路”战略。“辽（营）满欧”大陆桥累计开通中欧班列11条，全年完成集装箱运量3.3万标准箱，增长30.8%，占满洲里口岸出境量的51.3%。国际贸易“单一窗口”投入运行，营口港集装箱多式联运成为全国首批示范工程。港口货物吞吐量完成3.52亿吨，增长4%；集装箱运量608.6万标准箱，增长2.8%。

对外开放载体不断完善。积极推动跨境电商平台建设，营口港（荟）莫斯科海外仓投入使用，阿里巴巴全球货源平台营口站入驻企业225家。

对外交流合作实现新发展。“走出去”步伐加快，注册登记境外投资项目20个，新增投资7.5亿美元。罗马尼亚辽宁境外经贸合作园区规模不断壮大。营口港与俄铁公司合作持续深化，莫斯科别雷拉斯特物流中心项目已签约。

（五）统筹协调优环境，城乡面貌不断改善

城市规划管理水平稳步提升。坚持规划引领城市建设与发展，编制完成城乡发展“十三五”规划、新型城镇化规划和城市地下综合管廊等专项规划，城市规划体系逐步完善。深入开展文明城市创建活动，全面整治非法营运“三轮车”、马路市场等城市顽疾，城区秩序持续改善。

新老城区建设协调并进。围绕完善城市功能，加强基础设施建设。兰旗机场已通航上海、深圳、哈尔滨、济南4座城市，客流量突破11万人次。东海大街、镜湖西路建成通车，学府路拓宽等7项街路改造工程全面完工，盼盼路南端排水改造、泵站建设工程建成运行。沿海产业基地、营东新城配套设施日益完善，新城区综合承载能力进一步增强。

生态建设不断加强。严守生态红线，下大力气防治大气污染，全年拆除燃煤小锅炉865台，淘汰黄标车及老旧车15711辆，新增清洁能源公交车125辆。强化水污染治理，大旱河、沙河、熊岳河综合治理工作全面启动。持续推进生态环境修复，完成11个闭坑矿生态治理项目，人工造林3700亩，义务植树112万株，我市荣获“全国绿化模范城市”称号。

（六）尽心竭力惠民生，社会各项事业迈上新台阶

持续加大民生投入力度，全年重点民生支出达到一般公共预算支出的80%，去年确定的10件重点民生实事全部完成年度计划，营口获评“2016中国全面小康特别贡献城市”。

社会事业加快发展。

围绕优化公共服务和产品供给，抓急需、补短板。均衡发展各级各类教育，学前三年入园率达到96%，六县（市）区全部通过义务教育发展基本均衡县（市、区）省级评估验收。继续深化医药卫生体制改革，全科医生培养基地即将交付使用。实施文体惠民工程，为基层配备文化器材300余套、安装健身器材1240件（套），图书大厦主体工程竣工。实施食品药品安全专项整治，食品药品安全形势稳中向好。

就业和社会保障工作扎实推进。全面落实各项就业政策，全年城镇新增就业1.89万人，零就业家庭持续保持动态为零。城乡居民社会保障标准继续提高，企业退休人员养老金月人均增加135元，新农合和城镇居民医疗保险财政补助标准每人每年分别增加40元，城市低保、农村低保、农村五保标准分别提高5.5%、10.1%、7%。

社会治理进一步强化。深入推进普法和平安营口建设，我市被评为“全省法治宣传教育先进市”、“平安辽宁建设先进市”。健全矛盾纠纷调处化解综合机制，信访工作信息化、法治化和精细化水平全面提升。充分发挥“12345”服务热线等平台作用，一批热点、难点问题得到妥善解决。强化安全生产责任落实，全市安全生产形势持续平稳。

国防教育、民兵预备役和人民防空工作深入开展；我市荣获全国双拥模范城“四连冠”、省双拥模范城“六连冠”；统计、国家安全、民族宗教、史志、档案、气象、地震等工作不断加强；慈善、红十字、老龄、妇女儿童、残疾人等各项事业取得新成绩。

一年来，面对压力和考验，我们以开展“两学一做”学习教育为契机，认真落实全面从严治党的各项要求，始终坚持严守党的政治纪律和政治规矩，牢固树立“四个意识”，定期向市委汇报工作，全面落实市委的部署要求。自觉接受市人大及其常委会的法律监督和工作监督、市政协的民主监督，广泛听取各民主党派、工商联、无党派人士和各人民团体的意见。高度重视建议、提案办理工作，206件人大代表建议、282件政协提案全部办复。始终坚持依法行政，制定了法治政府建设实施方案。大力推进政务公开，严格执行重大行政决策规则，不断提高科学决策、民主决策水平。始终坚持从严治政，严格落实中央“八项规定”精神，认真做好中央巡视“回头看”反馈意见整改工作，强化行政监察、审计监督和督查督办，政府自身建设得到进一步加强。

各位代表：过去一年取得的成绩来之不易，这是省委、省政府和市委正确领导的结果，是市人大、市政协和社会各界监督支持的结果，是全市人民共同奋斗的结果。在此，我代表市政府向辛勤工作在各条战线的同志们，向各级人大代表、政协委员、各民主党派、工商联、无党派人士、各人民团体，向离退休老领导、老同志，向驻营解放军、武警部队官兵和中省直单位，向所有关心、支持营口建设和发展的同志们、朋友们表示衷心感谢并致以崇高敬意！

在肯定成绩的同时，我们也清醒认识到存在的问题。从经济运行看，下行压力仍然较大，去年地区生产总值、固定资产投资等指标没有完成全年计划任务，产业结构调整和转型升级成效还不明显；从社会和民生看，文教卫生、社会保障等基本公共服务供给水平还不够高，一些涉及群众利益的问题还没有切实得到解决；从风险隐患看，财政收支矛盾依然突出，民生保障、偿还债务、弥补养老金缺口等刚性支出不断增加，可用财力不足；从政府自身建设看，“放、管、服”改革还不到位，少数部门和个别干部责任意识、担当意识不强，只要权力、不要责任，不作为、慢作为、乱作为现象仍然存在，个别腐败问题严重损害了政府形象。这些问题需要我们勇于面对，深刻反思，认真解决。

各位代表：过去的一年，虽然我们付出了艰辛努力，但部分经济指标依然没有实现预期目标，这既有全球经济低迷、国内“三期叠加”、经济下行的影响，也有我们坚持全省一盘棋、实事求是做实经济数据的因素。

尽管部分经济指标没有完成计划，但我市经济总量在全省第四的位次没有变，经济发展长期向好的趋势没有变，持续增长的良好支撑基础和条件没有变，群众生活不断改善的进程没有变。同时，全市经济增长方式在转变、发展动力在转换、经济结构在调整，这些积极变化正在为营口的振兴发展积蓄力量。

各位代表：加快振兴发展、全面建成小康社会，虽然面临许多困难和挑战，但我们要看到，党中央实施新一轮东北老工业基地振兴战略，出台一系列推动东北振兴发展的政策举措，含金量之高、支持力度之大前所未有，政策效应日益凸显；我们要看到，省第十二次党代会选举产生了新的坚强领导集体，描绘了全省今后五年的宏伟蓝图，发展思路清晰可见，为营口振兴发展进一步指明了方向、增添了动力；我们更要看到，营口有扎实的发展基础、活跃的民营经济、充沛的要素资源、良好的营商环境，改革红利、市场活力、发展潜力正在逐步释放。更难能可贵的是，营口政治生态正在得以修复，全市广大干部精神状态好、干事劲头足、工作热情高，为我们应对挑战、战胜困难提供了坚强保障。同时，随着国家“一带一路”战略的深入实施，特别是辽宁自贸区获批，更为我们抢占新一轮对外开放制高点、提升开放发展层次与水平创造了绝佳机遇，提供了强大动力。

信心就是希望、信心就是力量。只要我们胸怀梦想，咬紧牙关不放松；只要我们矢志坚持，撸起袖子加油干，就一定能够战胜前进中的任何艰难险阻，就一定能够打赢营口改革开放的攻坚战，就一定能够开创营口全面发展的美好未来！

二、2017年工作安排

今年是实施“十三五”规划的关键一年，是新一轮开放发展的突破之年，做好今年工作意义重大。

今年政府工作总的要求是：以党的十八大和十八届三中、四中、五中、六中全会及中央经济工作会议精神为指导，深入学习贯彻习近平总书记系列重要讲话精神，按照“五位一体”总体布局和“四个全面”战略布局要求，践行“五大发展理念”和“四个着力”，落实省委、省政府和市委的决策部署，坚持稳中求进工作总基调，以推进供给侧结构性改革为主线，以提高发展质量和效益为中心，抢抓机遇，优化环境，强化项目建设，补齐民生短板，全力以赴促进全市经济社会平稳健康发展，以优异成绩迎接党的十九大胜利召开。

今年全市经济社会发展的主要预期目标是：地区生产总值增长7%，固定资产投资增长13%，一般公共预算收入增长5.5%，社会消费品零售总额增长9%，城乡居民收入增长与经济发展同步，城镇登记失业率控制在3.7%以内。

为实现上述目标，重点抓好以下七项工作：

（一）以项目建设为中心，增强经济发展新动能

坚持把项目建设作为头等大事，围绕项目抓发展，动员一切力量，发挥一切优势，坚定不移抓招商上项目。

持续优化营商环境。牢固树立抓营商环境就是抓经济发展的强烈意识，制定出台优化营商环境实施意见，集中开展专项整治行动，充分运用新闻监督、群众监督等监督手段，建立健全奖惩机制，打一场改善营商环境的攻坚战。坚决扭转“庸、懒、散、慢、乱”的不良风气，坚决破除不托人办不了事、不给钱不办事的“潜规则”，坚决杜绝不守信用、知法违法的行为。特别是各级政府要带头强化契约精神和法治思维，大力推进诚信营口、法治营口建设，守信践诺、法之必行，不断提升政府公信力。推进政务服务提速提质提效，实施保姆式服务，做到招商安商并重，构建“亲”“清”新型政商关系，努力打造法治化、国际化、便利化的营商环境。

加大招商引资力度。进一步完善招商引资工作机制，落实各县（市）区、园区和各部门招商引资责任，努力形成人人想招商、人人会招商的浓烈氛围。坚持内外资并重，引资引智引技并举，围绕重点产业，突出重点地区，加强与江苏对口合作，实施产业链招商、点对点招商、龙头企业招商，加快引进一批重大项目。高效利用存量资源，通过二次招商有效盘活长期未开工建设、未竣工投产的土地和项目，促进现有企业增资扩产，形成新增量。全年国内实际到位资金350亿元。

强力推进项目建设。坚持把项目储备放在更加突出的位置，做到一个部门、一个行业、一个项目库，重点围绕贯彻落实中央一系列东北振兴政策，聚焦系统、行业专项规划，全年包装亿元以上项目超过300个，总投资2000亿元以上。抓好招商引资和项目开工建设，做到一个项目、一个领导、一个团队、一抓到底，重点推进忠旺120万吨板带箔等226个亿元以上项目实现开工。促进项目投产运营，做到一个企业、一个部门、一个班子、一包到底，着力推进华能仙人岛热电厂等224个在建项目建成投产。全年固定资产投资完成464亿元，开工千万元以上项目超过400个。

（二）以发展实体经济为重点，推动三次产业协调发展

坚持把着力推进产业转型升级作为主攻方向，推动一二三产业同向发力，振兴实体经济。

转变农业发展方式。推进农业供给侧结构性改革，加快培育农业农村发展新动能。抓好“米袋子、菜篮子”工程，粮食生产稳定在69万吨，设施蔬菜产量实现65.6万吨。大力发展现代高效农业，推动一产接二连三，延长农产品产业链。积极实施品牌提升计划，狠抓农产品标准化生产，增加优质农产品供给。着力抓好县域经济发展，培育特色优势产业。继续深化农村综合改革，完成30.5万亩耕地确权登记颁证任务，推进农村土地“三权分置”。培育新型农业经营主体和服务主体，发展多种形式的适度规模经营，充分释放农村发展活力。发展农村普惠金融，切实解决农民“贷款难”问题。

推动工业结构优化调整。坚持做强存量、做优增量、做大总量，打造工业经济升级版，努力建设制造强市。深入落实《中国制造2025营口行动纲要》，重点推进10个国家和省级试点示范及50个市级重点支撑智能制造项目建设。落实国家、省关于培育和发展战略性新兴产业的部署要求，推动新材料、节能环保、海洋装备、海洋生物等新兴产业发展。继续开展“千企转型升级行动”，加快利用新技术新业态改造提升冶金、石化、镁质材料等传统产业，推动企业提质增效。精准帮扶困难企业，促进工业经济平稳运行。全年规模以上工业增加值增长7.5%。

促进服务业提档升级。围绕发展大物流、大商贸、大旅游，打造现代服务业发展新格局。依托综合交通网络，大力发展现代物流业，打造物流分拨中心，加快营口港中俄国际集装箱物流园等项目建设，培育一批大型专业贸易商及物流企业。积极引进研发中心、咨询中心、评估中心等生产性服务业，与大型知名电商寻求合作，争创国家电子商务示范基地。提升传统商业发展水平，推进实体店网上商城建设，实现线上线下融合发展。不断壮大金融业，推动金融创新，引进金融机构5家，金融业增加值增长8%。采取切实有效的政策措施，推动信息、健康、养老、教育、体育、文化等新型服务业态实现新突破。加快发展旅游业，以“四大景区组团”为核心，开发旅游产品，打造旅游品牌，扶持旅游企业做大做强，努力把旅游业打造成为支柱产业。整合辽河老街、西炮台等旅游资源，加快建设主城区西部特色旅游带。以鲅鱼圈区创建国家全域旅游示范区为牵动，完善景区基础设施和管理服务，加速发展全域旅游。全年旅游总收入实现235亿元。

（三）以供给侧结构性改革为主线，激发内生发展动力

坚持把着力完善体制机制作为治本之策，以改革破除体制机制障碍，为经济社会发展注入新动力。

深入推进供给侧结构性改革。通过深化改革，推动建立市场取向的体制机制，充分发挥价格机制的资源配置作用，健全激励创新机制，引导企业适应市场需求，不断改进产品和服务质量。做好“三去一降一补”。鼓励和引导企业淘汰落后产能，全面完成省下达的去产能任务。释放城镇居民改善性住房需求，引导农村居民进城，鼓励域外人员购房，规范发展住房租赁市场，打通供需通道，房地产去库存200万平方米，完成7422套棚户区住房改造任务。把降低企业杠杆率作为重中之重，依托股权（创业）投资引导基金和中小企业可持续发展基金撬动更多社会资本投资，支持企业上市融资。落实国家优惠政策，严格执行收费清单管理，降低各类制度成本、评估费用、用能成本、物流成本，促进企业降本增效。从制约经济社会发展的重要领域和关键环节、从人民群众迫切需要解决的突出问题着手，补齐创新创业、公共服务、民生保障、生态环境等发展短板和制度短板。

持续深化“放、管、服”改革。全面推行“互联网+行政审批”和“一窗受理、合并审查、集中审批、一站办结”的智能化审批服务模式，实现“一枚印章管审批”，压缩审批时限。强化事中事后监管，大力推行“双随机、一公开”监管制度，实现市场监管执法事项随机抽查全覆盖。实行首问负责、全程代办、容缺审批、限时补办等系列优化服务措施，探索联合执法机制，为市场主体创造宽松的经营环境。继续推进商事制度改革，全面推广个体工商户“两证整合”登记制度。

着力实施财政和投融资体制改革。加强政府性基金预算、国有资本经营预算与一般公共预算统筹，实行零基预算，提高预算执行约束力。加强支出管理，集中开展“五清理”工作，堵塞支出漏洞。积极防范债务风险，通过国有企业集团划转等途径化解政府债务，确保债务不出现风险。加大政府和社会资本合作模式实施力度，重点在基础设施、环保、能源等领域推进一批项目。继续推动企业拓宽直接融资渠道，新增5户企业进入“新三板”以上资本市场，新增融资总量超过500亿元。

积极开展国资国企和事业单位改革。以管资本为核心，创新国有资产监管方式，建立更具活力的企业经营机制，坚定不移做强做优做大国有企业。推动竞争类国有企业实行股份制改造，探索引进社会资本与供水、供热、燃气等公益性企业开展合资合作。妥善解决国有厂办大集体等历史遗留问题。坚持市场化改革取向，以“转”为主、撤并结合，将具备条件的生产经营类事业单位转制为企业或组建企业集团。

大力支持民营经济发展。全力实施国家东北地区民营经济发展改革示范城市建设，重点在政策环境、创新环境、市场环境建设和促进民营经济转型升级领域开展示范工作，为民营企业创新发展松绑减负，提供周到服务。健全融资、担保等服务平台，支持中小微民营企业发展壮大。

（四）以“一带一路”建设为牵动，构筑对外开放新高地

坚持把对外开放作为营口实现新发展的不竭动力，不断扩大对外开放，推动外向型经济发展再上新台阶。

抢抓开放新机遇。以辽宁自贸区建设为契机，充分依托港口、区位优势，以自贸区思维和平台谋划工作，不断改进和创新体制机制，开创营口开放发展新局面。加快综合保税区申办及建设步伐，搭建对外开放新平台。立足东北亚开放合作，结合营口实际，加快发展国际贸易、跨境电商、产业金融、总部经济、信息技术等重点产业，努力建设国际海铁联运大通道、国际区域性商贸物流和转口贸易中心、高端装备制造和高新技术产业基地。

深度融入“一带一路”。以“辽（营）满欧”大陆桥与辽鲁陆海大通道为依托，积极对接中蒙俄经济走廊，强力推进营口港集装箱多式联运示范工程建设，形成辐射东北亚、联通中东欧的大物流格局，努力把营口发展成为中国向北开放的重要窗口。支持营口港转型升级、做大做强，加快推进仙人岛港区2号30万吨原油码头等港口重大项目建设，进一步完善港口功能。坚持以港兴市战略，完善港城联动政策，力争在港城融合发展上实现新突破。

提升重点园区发展水平。以提质增效升级为核心，推动园区实现绿色集约发展。围绕项目落地，加快完善基础设施，提升园区承载能力。进一步强化园区经济发展职能，充分发挥园区在吸引投资、集聚产业方面的主要载体作用，重点抓好招商引资和项目建设，为企业建设运营提供优质高效服务。不断完善发展规划，防止低水平重复建设。实行差异化发展战略，避免同质竞争。理顺各园区和所在行政区管理体制，充分调动县（市）区和园区两个积极性。

扩大对外贸易。用足用好外贸扶持政策，创新对外贸易体制机制，激发对外开放新活力。积极吸引进出口企业在营注册，引导企业调整产品结构，做大进出口规模。加大“走出去”力度，鼓励企业赴外开展工程承包带动出口。推进口岸单位通关协作，促进贸易便利化。加快中韩投资贸易合作园区建设，打造韩国商品进入东北、内蒙古、中东欧的贸易集散地。外贸出口占全省比重达到7.8%。

（五）以创新驱动为引领，打造经济发展新引擎

坚持把着力鼓励创新创业作为动力支撑，深入实施创新驱动发展战略，推动以科技创新为核心的全面创新，让创新成为振兴发展的主动力。

强化企业创新主体地位。支持企业独立或联合高校、科研院所，建立省市级工程技术研究中心和产业技术创新战略联盟20家以上。推进核心技术和配套技术的研发攻关，促进科研成果产业化。全年实施科技成果转化项目30项，有效发明专利增长10%。

完善科技创新服务体系。开展科技成果库、技术需求库和专家人才库建设，发挥科技创新网络综合服务平台和科技文献共享平台作用，为企业提供便捷、高效服务。继续引进省内外高校和科研院所技术转移机构，探索建立统一的专利技术信息服务平台，推动专利技术在营转让转化。支持科技服务业发展。推动大众创业、万众创新，加大营口智能产业园等创业孵化平台建设力度，开展“双创”科技信息服务活动。

培育科技人才队伍。紧紧围绕我市发展需求，积极引进创新型领军人才和高水平创新团队，推进院士专家工作站建设，培育高技能人才队伍，激发人才创新创业活力。更加重视科技企业家人才，优化企业家创业成长环境，建设高素质企业家队伍。

实施创新政策引领。进一步完善高新区体制机制，为全市科技创新提供强力支撑。全面落实和完善促进科技创新发展的政策，加强对知识产权保护和高层次人才引进的政策引导，加快实现创新政策对创新链的全覆盖。继续实施创新券补助政策，支持中小微企业发展。

（六）以“美丽营口”建设为主题，不断优化城乡环境

坚持人民城市为人民，加强城市建设，提高城市治理能力，努力把营口建设成为功能完备、城乡和谐、生态宜居的海滨城市。

建管并重提升城市品位。探索多规合一，统筹考虑城市功能定位和空间布局，推动城市协调、可持续发展。加强城市建设和改造，对410路、新华路、平安路北段进行整修，对渤海大街、市府路、盼盼路等15条主要街路临街建筑实施景观亮化改造提升。深度挖掘城市文化底蕴，打造营口独特城市文化，提升历史文化街区和民俗民间文化内涵，支持辽河老街非物质文化遗产展示馆建设，筹建雷锋文化展览馆。推进交通、城管指挥中心数字化平台建设，提升城市精细化管理水平。盘活沿海产业基地、营东新城现有各类资源，完善生产生活服务设施，集聚人气，焕发新区活力。加大对违法建设、马路市场、露天烧烤以及乱停车、乱占道等行为的整治力度，逐步实施垃圾分类，加强小区物业、公共设施管理，优先发展城市公交，妥善解决交通拥堵等人民群众反映强烈的问题，为百姓营造良好的生活环境。

因地制宜建设美丽宜居乡村。以改善环境为突破口，坚持规划引领、整治先行，加强县城和重点乡镇建设，进一步完善县城功能、提升形象。继续加强宜居乡村和美丽乡村建设，重点解决垃圾处理、污水处理、秸秆综合利用等问题，推进畜禽养殖废弃物处理和资源化利用，完成8个宜居示范乡（镇）、40个宜居示范村和30个美丽乡村示范村建设任务，使广大农民生活得更加舒适、幸福。

持续加强环境保护和生态建设。坚持绿水青山就是金山银山的绿色发展理念，强化环境监管治理，加大环保执法力度，严肃查处环境违法行为，坚决守住生态红线。实施蓝天工程，狠抓扬尘、燃煤等污染源治理，推进冬季清洁取暖，拆除燃煤小锅炉302台，淘汰黄标车及老旧车6795辆。推进碧水工程，以大清河等综合治理为重点，实施入海河流集中整治和生态景观修复提升工程，启动北部污水处理厂建设，加快永远角湿地垃圾填埋厂整治，切实改善我市水环境质量。抓好青山工程，全面完成省下达的闭坑矿、生产矿生态治理任务。巩固国家森林城市创建成果，全年人工造林3600亩，义务植树100万株，封山育林1000亩。开展沃土工程，抓好污染土壤治理修复，严控新增土壤污染。环境污染是民生之患、民心之痛，必须铁腕治理；生态环保贵在行动，成在坚持，必须久久为功。我们一定要不懈努力、持之以恒，努力实现蓝天常在、绿水长流、永续发展！

（七）以民生改善为目标，不断提升群众幸福指数

坚持把着力保障和改善民生作为根本目的，政府再困难也要保民生投入，也要保基本、兜底线，实施好24件重点民生实事，进一步织密扎牢民生保障网，让全市人民共享振兴发展成果。

积极推动就业创业。着力抓好高校毕业生、退役军人就业创业工作，全年城镇新增就业1.8万人，高校毕业生就业率达到90%以上，零就业家庭继续保持动态为零，确保全市就业形势总体平稳。

促进城乡社会保障普惠公平。规范社保基金征缴和审批发放流程，积极筹措资金弥补养老金支出缺口，确保基本养老金按时足额发放。推动城乡居民医保制度整合，继续适当提高基本养老金等社会保障标准和城乡居民基本医疗保险政府补助标准。加强社会救助，保障特困群众基本生活。完善养老服务体系，推广社区居家养老，实施公办城市社会福利机构、农村敬老院维修改造，提高服务质量。强化困境儿童保障，努力让每一名儿童享受到幸福无忧的生活。

提升公共服务均等化水平。深化各级各类教育改革，推进城乡义务教育一体化发展，统一城乡“两免一补”政策，通过加强课堂教学考核等措施全面提升教育质量。完成市第一中学搬迁改建，推进营口职业技术学院、营口中专、市特殊教育学校建设改造，支持营口理工学院建设应用型大学。深化医药卫生体制改革，以医师多点执业为突破口，健全城乡医疗卫生服务体系，建立分级诊疗制度。发挥医保基金在资源配置和费用控制中的作用，引进社会资本参与公立医院改革。加快构建现代公共文化服务体系，推进市群众艺术馆、博物馆新馆建设，完善提升市图书馆、少儿图书馆新馆服务功能。大力发展体育事业，普及全民健身运动。强化食品药品和农产品质量安全监管，确保人民群众饮食用药安全。

深入实施东部振兴战略。大力扶持水果、蔬菜等主导产业发展，积极开发乡村旅游、沟域经济。加强农田水利等基础设施建设，加快农村社会事业发展，不断改善农村生产生活条件。继续实施脱贫攻坚，全年稳定脱贫13700人、贫困村摘帽10个。

加强和创新社会治理。广泛开展文明城市等群众性精神文明创建活动，不断提高市民素质。建设立体化社会治安防控体系，严密防范、依法惩治各类违法犯罪行为。扎实推进“七五”普法，实施法律援助惠民工程。落实信访工作责任，认真开展领导干部下访包案和积案化解等工作，依法解决群众合理诉求。严格落实安全生产责任，完善安全监管体制，强化依法治理，不断提高安全生产水平，努力建设安定有序的和谐城市。

继续推进国防教育、民兵预备役建设，支持驻营部队改革和军民融合发展，积极开展双拥共建活动。做好统计、人民防空、国家安全、民族宗教、史志、档案、气象、地震等工作。积极发展慈善、红十字、老龄、妇女儿童、残疾人等各项事业。

各位代表：面对新形势、新任务、新挑战，我们必须牢固树立“四个意识”，坚决维护以习近平同志为核心的党中央权威和集中统一领导，自觉在思想上政治上行动上与党中央保持高度一致，坚决服从省委、省政府和市委的领导，切实把全面从严治党各项要求贯穿于政府工作全过程，着力抓好政府自身建设，努力建设人民满意的政府。

我们要坚持勤政为民。强化宗旨意识，突出以人民为中心的发展思想，始终把人民群众的冷暖放在心上，从解决人民群众普遍关心的突出问题出发，推进全面小康社会建设。真诚倾听群众的呼声和诉求，虚心接受群众和社会各方面的监督，真正把老百姓的事情办实、办好。

我们要坚持依法行政。认真执行市人大及其常委会的决议、决定，坚持重大事项向人大报告、向政协通报制度，主动接受市人大及其常委会法律监督、工作监督和市政协民主监督，认真听取各民主党派、工商联、无党派人士和各人民团体的意见。严格依法行使权力，完善权责一致、执行顺畅、监督有力的行政管理体制。依法履行重大决策法定程序，严格执行“三重一大”制度，促进科学决策、民主决策。

我们要坚持务实高效。大力弘扬求真务实、埋头苦干的工作作风，遇到问题不退缩畏难、不等待观望、不推诿扯皮，敢于担当、敢于负责。健全激励约束和容错机制，解决不作为、慢作为、乱作为的问题。我们既要为担当者担当、为负责者负责、为干事者撑腰，又要对不担当不作为的进行严肃追责。加强学习，提高本领，特别是提升创造性开展工作的能力，真正将理论、政策、经验与营口实际结合起来，确保工作措施管用有效。持续不断提高工作效率，凡事定了就办、办就快办、办就办好。任何事情都要抓小抓细抓实，抓责任人抓节点抓效果，钉钉子钉到底，坚决反对“忽悠”。把精益求精、准确无误、细致入微、严格规范的要求贯穿到决策、执行、监督等各个环节，全面提升政府科学化、精细化管理水平。

我们要坚持廉洁从政。始终把纪律和规矩挺在前面，全面加强政府系统党风廉政建设，深入贯彻落实中央“八项规定”精神，坚决纠正侵害群众利益的不正之风。推进惩治和预防腐败体系建设，突出重点领域风险防范，加强行政监察和审计监督，将不敢腐、不能腐、不想腐的防线铸成铜墙铁壁。

各位代表：时代赋予重任，拼搏铸就辉煌。让我们紧密团结在以习近平同志为核心的党中央周围，在省委、省政府和市委的正确领导下，坚定信心，团结一致，勇往直前，攻坚克难，为不断开创营口全面发展新局面而努力奋斗！
